# Applications of CRISPR/Cas9 for the Treatment of Duchenne Muscular Dystrophy

**DOI:** 10.3390/jpm8040038

**Published:** 2018-11-24

**Authors:** Kenji Rowel Q. Lim, Chantal Yoon, Toshifumi Yokota

**Affiliations:** 1 Department of Medical Genetics, Faculty of Medicine and Dentistry, University of Alberta, 8812-112 St., Edmonton, AB T6G 2H7, Canada; kenjirow@ualberta.ca (K.R.Q.L.); cyoon@ualberta.ca (C.Y.); 2 The Friends of Garret Cumming Research and Muscular Dystrophy Canada HM Toupin Neurological Science Research Chair, 8812-112 St., Edmonton, AB T6G 2H7, Canada

**Keywords:** Duchenne muscular dystrophy (DMD), CRISPR/Cas9, exon skipping therapy, gene editing, human induced pluripotent stem cells (hiPSCs), immortalized patient muscle cells, *mdx* mice, humanized dystrophic mouse models, deltaE50-MD dog model

## Abstract

Duchenne muscular dystrophy (DMD) is a fatal X-linked recessive neuromuscular disease prevalent in 1 in 3500 to 5000 males worldwide. As a result of mutations that interrupt the reading frame of the *dystrophin* gene (*DMD*), DMD is characterized by a loss of dystrophin protein that leads to decreased muscle membrane integrity, which increases susceptibility to degeneration. CRISPR/Cas9 technology has garnered interest as an avenue for DMD therapy due to its potential for permanent exon skipping, which can restore the disrupted *DMD* reading frame in DMD and lead to dystrophin restoration. An RNA-guided DNA endonuclease system, CRISPR/Cas9 allows for the targeted editing of specific sequences in the genome. The efficacy and safety of CRISPR/Cas9 as a therapy for DMD has been evaluated by numerous studies in vitro and in vivo, with varying rates of success. Despite the potential of CRISPR/Cas9-mediated gene editing for the long-term treatment of DMD, its translation into the clinic is currently challenged by issues such as off-targeting, immune response activation, and sub-optimal in vivo delivery. Its nature as being mostly a personalized form of therapy also limits applicability to DMD patients, who exhibit a wide spectrum of mutations. This review summarizes the various CRISPR/Cas9 strategies that have been tested in vitro and in vivo for the treatment of DMD. Perspectives on the approach will be provided, and the challenges faced by CRISPR/Cas9 in its road to the clinic will be briefly discussed.

## 1. Introduction

Duchenne muscular dystrophy (DMD) is a severe neuromuscular disorder occurring in 1 in 3500–5000 male births worldwide that is due to recessive mutations of the *DMD* gene on the X chromosome [1,2]. This causes an absence of dystrophin, a protein essential for linking the actin cytoskeleton to the extracellular matrix in muscle cells, thereby providing membrane integrity [3,4,5]. As a result, DMD patients suffer from progressive muscle weakness, with the first symptoms presenting as early as 3–5 years of age [6,7]. Elevated levels of creatine kinase are typically observed in patient sera from birth [7,8]. Patients are also characterized by delayed motor, and in some cases cognitive, development [9]. Eventually, patients experience cardiac or respiratory failure during the third decade of life due to continued muscle degeneration, which ultimately results in premature death [10]. There is no effective treatment for DMD at present. Current strategies used in the clinic are focused on disease management, and mainly slow down progression at best.

The *DMD* gene spans 79 exons [11], and produces a full-length 427 kDa dystrophin protein [12] composed of four major domains: the N-terminal actin-binding domain, the central rod domain (containing 24 spectrin repeats), the cysteine-rich domain, and the C-terminal domain [13]. The latter two domains are primarily responsible for mediating interactions with associated proteins at the sarcolemma. The majority of DMD patients have large deletion mutations in the *DMD* gene (~60% of patients), the rest having duplications or point mutations [14]. Most mutations disrupt the *DMD* reading frame, typically creating a premature stop codon that prevents the translation of full-length dystrophin.

It has been observed that patients carrying in-frame as opposed to out-of-frame mutations in *DMD* usually present with a less severe disorder called Becker muscular dystrophy (BMD) [15]. This inspired the development of an approach known as exon skipping which aims to convert out-of-frame to in-frame mutations in DMD patients, with the expectation that this will lead to a milder phenotype. Exon skipping has commonly been accomplished through the use of antisense oligonucleotides (AOs), nucleic acid analogs that bind critical splice sites and/or exonic splicing enhancer elements to exclude (an) out-of-frame exon/s from the final mRNA transcript [16,17]. While the approach has experienced extensive development through the years [17], for instance with the development of various antisense chemistries, it remains a transient form of therapy, with patients requiring repeated administrations for sustained treatment. This can grow quite costly in the long run, given how expensive antisense drugs are. There is also the issue of delivery: AOs usually exhibit poor uptake to target tissues, particularly the heart [18,19]. This drastically reduces exon skipping efficacy in vivo, which lowers the therapeutic potential of the approach.

To help overcome this, clustered regularly interspaced short palindromic repeats (CRISPR)-based technology has been adapted in recent years for exon skipping in the context of DMD. When used in combination with associated enzymes such as Cas9 (CRISPR-associated protein 9), CRISPR allows for the targeted editing of virtually any specific sequence in the genome [20]. By strategically targeting certain *DMD* gene sequences for editing, splicing sequences can be altered/disrupted to classically skip exons in a similar manner as AOs. CRISPR can alternatively be employed to generate single or multiple *DMD* exon deletions [21], which essentially achieves the same reframing goal as exon skipping. Unlike AOs however, a distinct advantage of using CRISPR systems for DMD therapy is that they induce permanent corrections to the *DMD* gene and thus would not require repetitive and extensive durations of treatment. Also, as CRISPR components are typically delivered using viral vectors, with their own respective tissue tropisms, this increases the efficiency of delivery compared to AOs, and hence their activity in target tissues especially in relatively inaccessible areas such as the heart.

This review summarizes the studies and recent advances in the field that have employed the CRISPR/Cas9 system for DMD treatment. Although this approach can correct *DMD* patient mutations through a variety of ways, we will be focusing here on those that involve exon skipping through either classical/indirect (splice site disruption) or direct (exon deletion) methods. Non-exon skipping strategies such as the use of homology-directed repair (HDR) for gene correction and utrophin upregulation, among others, will be briefly touched on. We will begin with an overview of the mechanisms behind CRISPR/Cas9 gene editing, followed by a brief survey of in vitro and in vivo work relevant to the topic. We will then conclude with a discussion of issues, future directions, and alternatives for the approach.

## 2. Theory of CRISPR Application for Gene Editing

CRISPR/Cas9 gene editing was adapted from endogenous mechanisms of bacterial adaptive immunity against viral infection [22]. Bacteria capable of such activity possess CRISPR loci that contain three basic elements: the CRISPR array, the Cas9 enzyme gene, and the *tracrRNA* (*trans-activating CRISPR RNA*) gene [20]. The CRISPR array is composed of spacer and repeat elements interspersed with one another. The spacer elements are derived from pieces of foreign viral DNA that a bacterium processes say, upon an infection. Transcription of this array produces pre-CRISPR RNAs (pre-crRNAs), which then hybridize with tracrRNAs produced from the same loci via base pairing at the repeat elements. This initiates enzymatic processing and maturation of the pre-crRNAs into crRNAs [20,23]. These tracrRNA-crRNA hybrids can now be recognized and bound by Cas9, an endonuclease that induces blunt-ended DNA double-strand breaks (DSBs). Cas9 is then guided to its target, a 20-nucleotide sequence that must be next to a protospacer adjacent motif (PAM) sequence, by virtue of complementarity with the crRNA it carries [24,25,26]. Cas9 is therefore an RNA-guided DNA endonuclease. In this manner, foreign DNA coming from infecting viruses is effectively degraded, preventing the progress of infection.

The process we have just described is that for type II CRISPR systems, and this is the system most commonly adapted for use in research at present. Other CRISPR system types (I, III–VI) are defined mostly by the number and kind of Cas enzymes required for their function [20]. For research purposes, the type II CRISPR system is simplified in that the crRNA and tracrRNA are fused together in one continuous molecule called the guide RNA (gRNA) [20,27]. Cas9 enzymes from various bacteria are also used, the most common being from *Streptococcus pyogenes* (SpCas9) or *Staphylococcus aureus* (SaCas9), and these are mainly distinguished based on the PAM sequence they recognize for target recognition, among other characteristics [28] (Table 1).

**Figure 1:**
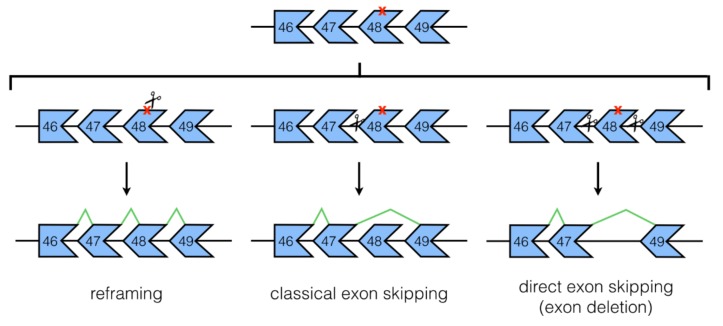
Mechanisms of non-homologous end joining (NHEJ) mediated gene correction by CRISPR/Cas9. On the top is shown a stretch of the *DMD* gene from a hypothetical patient with a point mutation in exon 48 (marked by an X) that creates a premature stop codon. CRISPR/Cas9, through NHEJ repair, can correct this genetic defect in one of three ways: reframing, classical exon skipping, or direct exon skipping. These approaches differ depending on which site is targeted by the gRNA (represented by scissors), as well as by the number of gRNAs used for treatment. Boxes represent exons; lines between boxes represent introns.

It is now therefore easy to visualize how the CRISPR/Cas9 system can be adapted for use in the precise editing of genomes. gRNAs can be designed to induce targeted DSBs in any select DNA sequence upon administration with Cas9, provided the target sequence is near a PAM site recognized by the Cas9 enzyme used [29]. Most of the time, this DSB is repaired by non-homologous end joining (NHEJ) mechanisms, which leads to random insertions and/or deletions (indels) at the site of cleavage. In the context of DMD treatment, this repair mechanism can generally be used in three different strategies (Figure 1). Firstly, if a single gRNA is used to target cleavage at or near a premature stop codon in a mutant or frameshifted *DMD* exon, indel formation by NHEJ can eliminate the stop codon and/or restore the reading frame back to the normal configuration (NHEJ reframing). Secondly, if a single gRNA is used to target cleavage at or near splicing sequences in *DMD* exons or introns, indels can disrupt these sites and allow for skipping of an out-of-frame exon to occur (classical exon skipping). And finally, if at least two gRNAs are used to target cleavage in separate exons or introns, deletions of one or more exons can be achieved to restore the *DMD* reading frame (direct exon skipping). Of particular interest in this review would be the second and third approaches mentioned. As we will see, these strategies, used alone or in combination with each other, have been used for developing various potential DMD therapies both in vitro and in vivo.

The DSBs created by CRISPR/Cas9 can also be repaired through another mechanism: HDR. In HDR, instead of randomly joining the two DSBs together, a template with ends homologous to either end of the DSB is used to precisely repair the lesion to make it have the same sequence it did before the DSB occurred [24]. However, as this occurs at much lower frequencies than NHEJ, and since it does not typically occur in post-mitotic cells [24], HDR is not used as often in DMD therapy development.

## 3. DMD Studies In Vitro and Alternatives to Viral Delivery of CRISPR

Table 2 comprehensively summarizes the studies that have thus far developed CRISPR strategies for the treatment of DMD. We recommend referring to this table as the succeeding sections of this review are read. Noticeably, the majority of these studies used the CRISPR/Cas9 system. As shown in the table, common strategies for editing the *dystrophin* gene in vitro involve the three NHEJ mechanisms described earlier. The potential of these approaches, as well as some unique others, have been demonstrated using a variety of in vitro models, including immortalized DMD patient muscle cells, primary DMD patient cells, *mdx* mouse satellite cells and, particularly, DMD human induced pluripotent stem cells (hiPSCs) and their derivatives.

Interestingly, the use of DMD patient-derived hiPSCs for the pre-clinical study of CRISPR/Cas9 therapies is currently gaining traction. hiPSCs can be derived from easily accessible patient tissues and, owing to their pluripotency, can be directed to differentiate into relevant cell types for CRISPR/Cas9 DMD therapy testing. Cardiomyocytes derived from hiPSCs can more accurately model the developmental progression and physiology of the human heart, which is insufficiently represented in the various animal models. For example, the cardiomyopathy shown by *mdx* mice is notably less severe than that observed in patients; also, the regulation of cardiac vasculature, ion channel activity, and myosin function are regulated differently in mice [33,34,35]. There is also the fact that mice and dogs often have increased tolerance and resistance to the cardiotoxic effects of drugs [36] and therefore may lead to an inaccurate representation of these drugs’ effects in humans. As alluded to earlier, hiPSCs also offer the advantage of reliably replicating patient mutations in cardiomyocytes in vitro without the need for invasively obtaining cardiac biopsies. Currently, cardiac muscle cells are obtained from transplants, irreversibly damaged hearts or through the transdifferentiation of fibroblasts; however, hiPSCs can be obtained from cells in the skin or urine (after reprogramming) [37,38,39], and are capable of modelling more than 7000 DMD mutation types [40]. While we mostly discussed the utility of hiPSCs for modelling the DMD phenotype in cardiac muscle cells here, groups have also differentiated skeletal myotubes from hiPSCs [41,42], offering somewhat similar advantages as described for iPSC-derived cardiomyocytes, particularly when biopsy samples are lacking for patients with certain mutations.

### 3.1. NHEJ Mechanisms: Classical and Direct Exon Skipping

Li et al. (2015) [41] demonstrated the potential of classical exon skipping with CRISPR/Cas9 for the first time in vitro. In their study, hiPSCs from a DMD patient harboring an exon 44 deletion were electroporated with plasmids for SpCas9 and a gRNA targeting the splice acceptor site for *DMD* exon 45. This resulted in successful exon 45 skipping by RT-PCR and restored dystrophin expression as evidenced by immunostaining and Western blot data. Li et al. used other strategies using the same gRNA as well such as NHEJ reframing of exon 45 and HDR knock-in of exon 44, which gave similarly positive results in terms of rescuing dystrophin production. Moreover, they also employed the use of transcription activator-like effector nucleases (TALENs), another gene editing system, to accomplish the same strategies above and found that it worked comparably well as CRISPR/Cas9.

Succeeding studies shifted their focus more towards the direct exon skipping application of CRISPR/Cas9; i.e., the generation of exon deletions. Ousterout et al. (2015) [21] provided foundational knowledge on the multiplexing capabilities of gRNAs, that two gRNAs used in tandem could effectively delete single or multiple exons in the *DMD* gene. Most strikingly, Ousterout et al. showed the feasibility of deleting the large exon 45–55 region using this multiplex approach, with gRNAs targeting *DMD* introns 44 and 55 electroporated with SpCas9 in immortalized muscle cells from a DMD patient with an exon 48–50 deletion. The genomic deletion of exons 45–55 was confirmed by sequencing and dystrophin rescue was confirmed by Western blot. The exon 45–55 region is considered a mutation hotspot for the *DMD* gene; it is estimated that skipping this entire region can treat ~47% of all DMD patients [16,67]. Furthermore, people lacking this exon 45–55 region in the *DMD* gene are typically asymptomatic or, at the very least, present with mild phenotypes [68]. Thus, this result showing the potential of CRISPR/Cas9-mediated for deleting the exon 45–55 region is highly promising. Notably, Young et al. (2016) [42] have also achieved exon 45–55 skipping using hiPSCs and their derivatives. Various groups have shown the feasibility of deleting other exons. Single deletions of exons 51 [21,60] and 53 [49,51] have been done, as well as deletions of the exon 3–9, 6–7, 7–11 [57], and 44–54 [49,51] regions in vitro.

At this point, it is important to recognize that one cannot just haphazardly skip or delete *DMD* exons, thinking that as long as the resulting product is in-frame means that the dystrophin protein produced from this would be functional. It has been shown that this is not always the case; in-frame deletions in patients can still result in DMD phenotypes [69]. This is strongly related to how well the structure of the dystrophin protein is preserved in these truncated forms of the gene/transcript. This concept is best illustrated by the study of Kyrychenko et al. (2017) [57], who tried three different multiple exon deletion strategies in DMD hiPSCs using CRISPR/SpCas9. Kyrychenko et al. (2017) found that different truncated forms of dystrophin yielded widely different functionalities upon in vitro testing in hiPSC-derived cardiomyocytes. For instance, cardiomyocytes with dystrophin produced from exon 7–11 deleted genes exhibited the least functionality in terms of calcium cycling, whereas those with dystrophin produced from exon 3–9 deleted genes functioned the best. This was explained by differences in protein stabilities of the produced truncated forms of dystrophin. In silico testing of planned exon skipping strategies is therefore highly recommended prior to the initiation of any pre-clinical work, to ensure that any produced truncated dystrophin isoforms have preserved functionality. This is all the more important when the strategy deals with the formation of “hybrid exons”, i.e., when NHEJ joins two internally-cleaved exons together, as was done by a number of groups [48,63].

CRISPR/Cas9 can also be used to correct duplication mutations of the *DMD* gene. In one study, Wojtal et al. (2016) [44] used a single gRNA against intron 27 to correct the *DMD* gene in primary patient fibroblasts with a duplication in exons 18–30. Because of the nature of the mutation, a single gRNA can be used in a multiplex approach, allowing for the deletion of multiple duplicated exons. Treated transdifferentiated myotubes exhibited 4.42% dystrophin protein of normal levels by Western blot, and restored expression of the dystrophin-associated glycoprotein complex (DAGC) protein α-dystroglycan. Using a similar concept, exon 2 [54] and exon 55–59 duplication mutations [61] have also been corrected in immortalized DMD patient muscle cells and DMD hiPSCs, respectively.

It should be mentioned that while our discussion here was mostly focused on exon skipping strategies, indel formation at mutation sites (e.g., premature stop codons) through NHEJ has also been used for reframing by itself. In fact, this is usually a secondary strategy observed in many studies that use classical or direct CRISPR/Cas9-mediated exon skipping. NHEJ reframing has been found to restore dystrophin protein production as well, but because it is highly mutation-specific, even more so than exon skipping, it is expected to have limited applicability in clinical practice.

### 3.2. Base Editing

The precise editing of single bases in a given sequence, without the involvement of any double-stranded DNA cleavage activity, is also possible using the CRISPR/Cas9 system. This is accomplished through the use of Cas9 enzymes that either have impaired nuclease activity, such as Cas9-D10A nickase or nCas9 [70] that can only make single-stranded DNA cuts, or are completely catalytically inactive—these latter Cas9 enzymes are called “dead” Cas9 or dCas9. These mutant Cas9 enzymes are fused with adenine or cytidine deaminases that can then induce base conversion [65,71,72,73]. Adenine deaminases convert A:T to G:C base pairs, whereas cytidine deaminases convert C:G to T:A base pairs. CRISPR/Cas9-mediated base editing for DMD treatment in vitro was demonstrated by Yuan et al. (2018) [66], who used an activation-induced cytidine deaminase (AID) enzyme fused to dSpCas9 or dSaCas9 in an approach they called “targeted AID mutagenesis” or TAM. In their work, Yuan et al. targeted the exon 50 donor splice site for base editing, eliminating the GT splice consensus sequence and thereby skipping exon 50 in DMD hiPSCs carrying a deletion in exon 51. Approximately 90% of the desired base editing was achieved at the genomic DNA level as revealed by high-throughput sequencing analysis when dead SaCas9-AID was used. Differentiation of corrected hiPSCs into cardiomyocytes showed dystrophin restoration by Western blot and immunostaining, decreased creatine kinase levels, suppression of miR-31 (a known inhibitor of dystrophin expression) expression, and rescue of β-dystroglycan expression by Western blot. Base editing has also been successfully performed in a DMD mouse model [65], a work which will be described in the later section on in vivo studies.

### 3.3. Utrophin Upregulation

Another avenue of DMD treatment is to upregulate the expression of utrophin in immortalized DMD patient muscle cells. Utrophin is a homolog of dystrophin that is expressed in the myotendinous and neuromuscular junctions (NMJs) of adult skeletal muscles [74,75]. Interestingly during fetal development, utrophin is localized at the sarcolemma of muscle cells, where it functions similarly as dystrophin in stabilizing the membrane [76]. It is eventually replaced later in development by dystrophin, at which point utrophin can no longer be found at the sarcolemma except at the NMJ. Because of its structural and functional similarity to dystrophin, various groups are attempting to upregulate or reactivate its expression in dystrophic muscle cells with the expectation that it can act as a dystrophin substitute [77]. Indeed, adenoviral delivery of utrophin in the golden retriever model of DMD has been shown to ameliorate the pathophysiology of the disease [78]. Utrophin upregulation has no adverse effects on non-muscle cells [79] and is a universal treatment strategy for all types of DMD mutations, making it an attractive therapeutic strategy.

Fortunately, the CRISPR/Cas9 system can be used in such a way as to induce utrophin upregulation. The approach would again entail the use of dCas9; however, in this case, it is instead fused to strong transactivator elements such as VP16. gRNAs can be designed to target specific gene promoters; these will guide dCas9-VP16 to the chosen sites where, through VP16, it can facilitate the recruitment of transcriptional activators that can then promote the expression of selected gene/s [80]. This approach was used by Wojtal et al. in 2016 [44], where they targeted dSpCas9-VP16 (with 10 tandem VP16 repeats) to the *UTRN* A and B promoters. Lentiviral vectors containing dSpCas9-VP16 and gRNAs were electroporated into immortalized DMD patient muscle cells harboring an exon 42–52 deletion. The group showed that utrophin protein amounts increased 1.7- to 6.9-fold post-treatment compared to non-treated cells, which led to the restoration of β-dystroglycan expression by Western Blot. Upregulation of *UTRN B* led to better increases in utrophin production than the upregulation of *UTRN A*. Although the mechanism behind this difference in effect is unknown, the fact remains that CRISPR/Cas9 can indeed be used to upregulate utrophin expression and can potentially compensate for dystrophin loss in DMD muscle cells. Future studies however certainly have to be conducted to further evaluate whether this CRISPR/Cas9 strategy can lead to functional benefit in vitro and, eventually, in vivo.

## 4. DMD Studies In Vivo

The first study considered to demonstrate the in vivo potential of CRISPR as a therapeutic approach for DMD is that by Long et al. in 2014 [43]. In this work, the researchers aimed to correct the nonsense mutation in exon 23 of the *Dmd* gene in *mdx* mice through NHEJ or HDR; a single gRNA was used to target the mutant exon. Components of the CRISPR/Cas9 system together with a single-stranded HDR template were injected into *mdx* zygotes at the 1-cell stage, followed by implantation of the zygotes into pseudopregnant mice. Genetically mosaic NHEJ- and HDR-corrected pups were obtained, with varying amounts of *Dmd* editing. Near wild-type levels of dystrophin expression were observed by immunostaining in pups with 83% NHEJ or 41% HDR correction of mutant *Dmd* at 7–9 weeks of age. More importantly, however, ~47–60% of muscle fibers showed dystrophin-positive staining in *mdx* mice with only 17% HDR correction. Corrected mice exhibited decreased serum CK levels and improved grip strength test results. Progressive restoration of dystrophin with age was also found for skeletal but not cardiac muscles, explained in part by the finding that skeletal muscle satellite cells had corrected *Dmd* genes following treatment.

The unique feature of this work by Long et al. [43] is that it showed the feasibility of CRISPR-mediated germline editing for DMD therapy. This approach, however, presents some difficulty for translation to human use not only due to technical challenges (e.g., post-mitotic cells exhibit low rates of HDR) but also because of associated ethical issues. It is noticeable that succeeding in vivo studies became more focused on developing somatic editing strategies that did not rely on CRISPR/Cas9-mediated HDR; e.g., exon skipping. The *mdx* mouse model for DMD and its many derivatives have been primarily used for these in vivo CRISPR/Cas9 studies. A canine DMD model is recently entering the scene [64], offering the alternative to test CRISPR/Cas9 therapeutics in a much larger, and more phenotypically-comparable, animal system.

### 4.1. Studies in DMD Mouse Models

Three studies, published back-to-back (-to-back) in the same issue of *Science* in January 2016 [45,46,47], best demonstrate the efficacy of CRISPR-induced exon deletion for DMD treatment in vivo. All three aimed to delete the mutant version of *Dmd* exon 23 from the genome of *mdx* mice. Adeno-associated virus (AAV) vectors (serotype 8 or 9) were used to deliver CRISPR/Cas9 components in vivo, either intramuscularly, intraperitoneally, or intravenously. Varying degrees of dystrophin restoration in both skeletal muscles and the heart was found in all studies via immunostaining and Western blot upon systemic treatment, accompanied by proper relocalization of DAGC members at the sarcolemma and improvements in skeletal muscle function. Little to no off-target effects were found with CRISPR/Cas9 treatment, as supported by sequencing or T7E1 analyses.

As discussed earlier, the deletion of single exons is a particularly limited approach for DMD treatment. Multiple exon deletion is more favorable, given how it increases therapeutic applicability to a greater number of DMD patients. Fortunately, the generation of multiple exon deletions has been shown feasible in vivo. Deletions of exons 21–23 [50], 52–53 [52], and the large 45–55 region [56] have been achieved thus far using DMD mouse models, with encouraging results. Particularly interesting are studies using humanized DMD mouse models, as this allows for in vivo efficacy testing of human *DMD*-targeting CRISPR/Cas9 strategies.

In 2008, ‘t Hoen et al. [81] generated transgenic mice which had a stable single-copy integration of a full-length human *DMD* transgene on chromosome 5. The various *DMD* isoforms showed similar tissue-specific expression in these humanized mice (hDMD mice) and, when hDMD mice were crossed to *mdx* or utrophin knockout *mdx* mice, the human *DMD* transgene was capable of rescuing the dystrophic phenotypes in these models. Young et al., in 2017 [56], sought to create a humanized dystrophic mouse model with this hDMD mouse as the starting point. They used CRISPR/Cas9 with gRNAs against human *DMD* introns 44 and 45 to delete exon 45 in hDMD mice (now called hDMD del45 mice), which results in an out-of-frame transcript. Indeed, crossing hDMD del45 mice to *mdx* mice on both the original C57BL/10 and DBA/2 backgrounds resulted in mice that lacked dystrophin expression and exhibited dystrophic histopathological phenotypes. Young et al. went a step further and, in the same study, used this new hDMD del45/*mdx* model to test the efficacy of their CRISPR/Cas9-mediated direct exon skipping strategy in vivo. They electroporated Cas9-gRNA plasmids into the flexor digitorum brevis (FDB) muscle of these mice, with the gRNAs targeting *DMD* introns 44 and 55 to generate an exon 45–55 deletion. The intended deletion was successfully observed in genomic DNA from treated FDB samples 22–33 days post-electroporation, and immunostaining revealed the presence of dystrophin-positive fibers. Iyombe-Engembe et al. (2016) [48] and Duchêne et al. (2018) [63] are studies which also used humanized DMD mice for in vivo testing of CRISPR/Cas9 strategies for DMD treatment, the former using hDMD/*mdx* mice and the latter using hDMD del52/*mdx* mice. Interestingly in the work of Duchêne et al. (2018), treatment resulted in restored dystrophin expression in the heart as observed by immunohistochemistry and Western blot; no quantification was performed, however. PCR with subsequent TIDE analysis in treated cardiac muscle samples revealed that CRISPR/Cas9 generated 83% or 86% of the expected correction, depending on the gRNAs used for the approach.

As alluded to earlier, base editing has also been performed in vivo as a potential treatment for DMD. Ryu et al. successfully demonstrated this in 2018 [65], using adenine base editor 7.10 (ABE7.10) [73] fused to nSpCas9 to correct the nonsense mutation in *Dmd* exon 20 of a DMD mouse model (which, interestingly, was created using CRISPR/Cas9 base editing [82]). In this work, the ABE7.10-nSpCas9 construct, owing to its large size, was split in half and delivered intramuscularly into the tibialis anterior (TA) muscle using a trans-splicing AAV vector system; the two parts would eventually combine via recombination at inverted terminal repeat sequences found in both constructs. Deep sequencing revealed an editing frequency of approximately 3.3% in the TA muscle 8 weeks post-treatment; no detectable off-target effects were observed. Treatment led to restoration of dystrophin expression as assessed by immunohistochemistry, with 17% dystrophin-positive fibers observed on average. This was coupled with restored localization of neuronal nitric oxide synthase, a DAGC member, at the muscle membrane.

While the majority of in vivo mouse studies have used either SpCas9 or SaCas9, other Cas enzymes have been used in conjunction with CRISPR-based DMD therapies. A good example of this is the study of Koo et al. (2018) [62], which used Cas9 derived from *Campylobacter jejuni* (CjCas9). The most highlighted feature of CjCas9 compared to other Cas9 enzymes is its size, being only 984 amino acids long and corresponding to a 2.95 kb gene (Table 1). This makes it smaller than either SpCas9 or SaCas9, which lends CjCas9 increased amenability to being packaged in viral vectors like AAVs. Furthermore, whole-genome sequencing studies show that CjCas9 has considerably higher targeting specificity than SpCas9, without a compromise in targeting efficiency [32]. This is likely due to the longer PAM site recognized by CjCas9, which also occurs at a lower frequency in the genome than that of SpCas9.

In their study, Koo et al. [62] use mice with an out-of-frame deletion in *Dmd* exon 23. They intramuscularly inject the TA muscles of these mice (8–12-week-old) with AAV9 vectors containing constructs of CjCas9 in tandem with a gRNA targeting the premature stop codon formed in exon 23. Treatment induced NHEJ indel formation, 27.2% of which corrected the *Dmd* reading frame. About 28 or 39% of dystrophin-positive fibers were observed by immunostaining depending on the nature of the generated indel. Treated mice also displayed significantly increased TA muscle strength (specific maximal force) than saline-treated mice. Thus, this study demonstrates that CjCas9 can also be used to develop CRISPR-mediated therapies for DMD, offering an attractive alternative to SpCas9 and SaCas9 in cases when viral packaging is the limiting step to therapeutic development.

### 4.2. Study in a DMD Dog Model

Only one study at present has ever applied CRISPR/Cas9 to treat a dog model of DMD. In a landmark study by Amoasii et al. (2018) [64], researchers used CRISPR/Cas9 to correct the genetic defect found in the deltaE50-MD canine DMD model. This model was created by bringing the intron 50 5′ splice site missense mutation in the dystrophin gene of Cavalier King Charles Spaniel dystrophic model dogs [83] into the beagle background. The described mutation causes the out-of-frame skipping of exon 50, which causes a lack of dystrophin. DeltaE50-MD dogs display histopathological phenotypes typical of DMD such as progressive fibrosis and fat accumulation; they also exhibit severe dystrophic degeneration of respiratory muscles [84]. The resemblance in phenotype to DMD patients makes this large animal model highly useful for in vivo studies testing CRISPR/Cas9 therapies.

Amoasii et al. (2018) [64] first intramuscularly deliver AAV9 vectors containing SpCas9 in tandem with an exon 51-targeting gRNA to the TA muscles of 1-month-old deltaE50-MD dogs (*n* = 2); treatment efficacy was evaluated 6 weeks post-injection. The gRNA used targets a sequence on exon 51 situated near the intron 50 splice acceptor site, which is expected to either induce indels that would reframe exon 51 (NHEJ reframing) or that would disrupt the acceptor site leading to exon 51 skipping—both restoring the reading frame and expected to lead to dystrophin restoration. Approximately 52 or 67% dystrophin of wild-type levels were observed in the TAs of treated dogs by Western blot; contralateral muscles injected with saline showed on average 2% dystrophin of wild-type levels. Improvements in histopathology and restoration of β-dystroglycan expression were found in the treated muscles as well. Immune response activation was not found, based on immunostaining data for CD4 and CD8 T cell markers. Deep sequencing revealed no significant editing on the most likely off-target sites as a result of treatment.

The researchers then systemically treated deltaE50-MD dogs by intravenously injecting them at 1-month old (*n* = 2, with one receiving a higher dose) with AAV9 vectors that separately contain the SpCas9 and gRNA constructs; analysis was conducted eight weeks post-injection. Dystrophin restoration was observed across various skeletal muscles and the heart by immunostaining. Quantitatively using Western blot, ~5–70% dystrophin of wild-type levels were observed in the different skeletal muscles while ~92% dystrophin of wild-type levels was observed in the heart of the dog given the higher treatment dose; the amount of dystrophin restored was correspondingly lower in the dog that received the lower dose. Histopathological improvements and restored expression of β-dystroglycan were likewise observed. Blood tests do not reveal any adverse effects of treatment.

While highly promising as a proof-of-concept, this study has several limitations. For instance, only two dogs were used at each phase of the study, with the second phase involving only one dog at each given dose, so consistency of effect cannot be implied from the results. The study was also not sufficiently long to allow for investigation of sustained therapeutic effect, which is one of the main goals of CRISPR-mediated DMD therapy, or of potential immunogenic responses upon systemic treatment. Finally, muscle function tests were not performed. These limitations are, however, understandable for a preliminary study of such scale. Hopefully, future studies can address these issues, so as to provide us with a better perspective of how CRISPR/Cas9 therapy will fare in a large animal model.

### 4.3. CRISPR/Cas9 for the Generation of DMD Animal/Cell Models

Not only has CRISPR/Cas9 been used to correct genetic defects in DMD animal models, it has also been used to accomplish the opposite; i.e., to deliberately induce permanent mutations in the dystrophin gene in vivo for purposes of creating new animal models of DMD. We have already described one such study that performed this, that of Young et al. (2017) [56], which described the creation of the hDMD del45/*mdx* model. Various groups have gone on to develop other DMD animal models using CRISPR/Cas9; at present, the list includes mice with other *Dmd* mutations [59,82], rats [85], pigs [86], rabbits [87], and monkeys [88]. In vitro models of DMD have also been created using CRISPR/Cas9, by generating *DMD* mutations in rhabdomyosarcoma cells [89,90] or in hiPSCs [57]. We will not be describing the characteristics of these models here, but instead gladly invite the reader to consult the cited references for additional information.

## 5. Conclusions

Before implementing CRISPR/Cas9 treatment clinically, more in vivo studies monitoring the off-target effects induced by the therapy and the potential immune responses activated by Cas9 or viral delivery vectors must be conducted. The unpredictability of gene editing is a great risk to safe clinical treatment, as undesirable mutations induced by CRISPR/Cas9 can occur in treated cells [91]. The extent to which CRISPR/Cas9 produces off-target effects is highly debated at present [92], with different studies presenting different rates of off-targeting. However, whichever extent it is, use of in silico tools [93] to predict potential off-targets is recommended during the gRNA design phase to minimize off-targeting as much as possible. Encouragingly for the field, CRISPR nucleases with greater fidelity are now being engineered to significantly reduce off-targeting in vitro and in vivo [94,95,96]. Modifications in the gRNA structure itself, e.g., changes in chemistry or length, have also been shown to improve CRISPR/Cas9 targeting specificity [97,98]. Such changes mentioned not only have beneficial effects for specificity, but also for efficacy. In terms of efficacy, however, one barrier that is increasingly becoming recognized is the p53-dependent DNA damage response induced by CRISPR/Cas9 treatment. It has been found that p53 activation as a result of double-stranded DNA break production by CRISPR/Cas9 leads to a decreased efficiency of gene repair in vitro [99,100]. Strategies to modulate p53 function may prove helpful in mitigating the negative effects of this phenomenon on the success of CRISPR/Cas9 therapy.

Immune response against Cas9 is also a challenge to therapeutic success [101,102]. This response may be partially induced as a result of previous exposure to the bacteria from which the Cas9 enzymes used were derived, which is fairly common for *S. pyogenes* and *S. aureus* [103]. As a consequence of this response, for example, immune effectors could eliminate Cas9-containing cells, which would render the therapy futile. While numerous strategies have been proposed to combat this [101], such as the engineering of Cas9 nucleases to decrease their immunogenic properties in vivo, these are still at the earliest stages of development and would take time until definitive results are produced. Research has identified that it may be possible to control this immune response using Cas9-reactive regulatory T cells; however, more work is required to determine whether such an approach is feasible and safe [103]. There is a long-standing issue on the immunogenicity of viral vectors used in gene therapy, which also must be addressed. Fortunately, steps are also currently being taken to resolve this important concern [60,104,105], which includes the development of non-viral delivery approaches such as nanoparticles [58].

Nonetheless, in contrast to AO treatment, CRISPR/Cas9 remains advantageous as it can permanently correct the genetic defect in DMD patients, which minimizes long-term treatment costs. Through a variety of approaches, we have seen how CRISPR/Cas9 systems have been successful in correcting the reading frame of the dystrophin gene in vitro and in vivo. CRISPR systems using enzymes other than Cas9 have also shown promising for DMD therapy. For instance, Cpf1 enzymes adapted from *Prevotella* and *Francisella* bacteria have been used to successfully reframe dystrophin exons in hiPSCs and in *mdx* mice [53]. Use of other Cas enzymes is expected to increase the number of CRISPR strategies developed for DMD therapy and could even reveal approaches of higher efficacy than when just Cas9 is used. One thing to note though is that a single CRISPR/Cas9 myoediting approach will not be a universally restorative treatment for all DMD mutations, so this has to be taken into consideration in the design of CRISPR strategies. CRISPR/Cas9 is indeed an innovative gene-editing tool, and has shown great potential to rectify DMD mutations. However, ongoing research is necessary to enhance the therapeutic safety and efficacy of CRISPR/Cas9 strategies in vitro and in vivo using animal models before it can enter clinical trials and finally be used to treat DMD patients.

## Figures and Tables

**Table 1:** Cas9 enzymes used in conjunction with clustered regularly interspaced short palindromic repeats (CRISPR) for Duchenne muscular dystrophy (DMD) treatment. Outlining the three Cas9 nucleases used thus far to reframe the *dystrophin* gene, and selected features.

Cas9 Enzyme	Source Bacteria	PAM Site	Protein, Gene Size	Key Features	Reference(s)
SpCas9	*Streptococcus pyogenes*	5′-NGG-3′	1368 aa, 4.10 kbp	Ubiquitous PAM site, widely used with multiples derivatives	[27,28,29]
SaCas9	*Staphylococcus aureus*	5′-NNGRRT-3′	1053 aa, 3.16 kbp	Smaller size, better packaged for viral delivery	[30,31]
CjCas9	*Campylobacter jejuni*	5′-NNNNACAC-3′, 5′-NNNNRYAC-3′	984 aa, 2.95 kbp	Even smaller than SaCas9, lower chance of off-targeting due to longer PAM	[32]

Abbreviation: PAM, protospacer adjacent motif.

**Table 2:** Summary of studies that have used CRISPR/Cas9 approaches for the treatment of DMD.

Cas Enzyme	Strategy	Target Gene Region(s)	Model(s)	Delivery	Study Highlights	Reference
SpCas9	NHEJ reframing, HDR exon correction	*Dmd* exon 23	*mdx* mice	1-cell embryo injection	Dystrophin restoration observed by IHC (up to 100%) and WB; 17% *Dmd* HDR correction resulted in 47–60% dystrophin-positive fibers in skeletal muscles and the heart	2014 Long et al. [43]
SpCas9	NHEJ reframing, exon skipping, HDR exon knock-in	*DMD* intron 44/exon 45	DMD hiPSCs, hiPSC-derived skeletal muscle cells (ex44 del.)	Electroporation	Dystrophin restoration in derived skeletal muscle cells observed by WB and IHC for all strategies; CRISPR was as effective as using TALEN	2015 Li et al. [41]
SpCas9	NHEJ reframing, single/multiple exon deletion	*DMD* exons 45–55 (for reframing each exon), introns 50 and 51 (ex51 del.), introns 44 and 55 (ex45–55 del.)	immortalized DMD patient muscle cells (ex48–50 del.), immunodeficient NSG mice	Electroporation	Generated targeted deletions of exon/s in vitro, particularly of the large exon 45–55 region which led to dystrophin rescue by WB; mice transplanted with treated myoblasts (exon 51-deleted) showed dystrophin-positive fibers by IHC	2015 Ousterout et al. [21]
dSpCas9-VP16	Utrophin upregulation	*UTRN* A/B promoter	immortalized DMD patient muscle cells (ex45–52 del.)	Electroporation	1.7–6.9-fold upregulation of utrophin achieved; restored β-dystroglycan expression observed by WB with as little as 1.7-fold upregulation	2016 Wojtal et al. [44]
SpCas9	Duplicated exons removal	*DMD* intron 27	primary DMD patient fibroblasts (ex18–30 dup.)	LV transduction, with Adeno-MyoD	4.42% full-length dystrophin production achieved post-treatment, accompanied with α-dystroglycan restoration	2016 Wojtal et al. [44]
SpCas9	Single exon deletion	*Dmd* exon 23, introns 22 and 23 (ex23 del.)	*mdx* mice	AAV9 delivery (i.m., i.p., i.v.)	All modes of injection led to appearance of dystrophin-positive fibers as evaluated by IHC: ~25.5% 6 wks post-i.m., ~4.6% and ~9.6% in skeletal and cardiac muscles respectively 12 wks post-i.v., ~1.8% and ~3.2% in skeletal and cardiac muscles respectively 8 wks post-i.p.	2016 Long et al. [45]
SaCas9	Single exon deletion	*Dmd* introns 22 and 23 (ex23 del.)	*mdx* mice	AAV8 delivery (i.m., i.p., i.v.)	Intramuscular injections led to ~59% of transcripts with exon 23 deleted, which restored about 8% dystrophin of healthy levels by WB, proper relocalization of DGC proteins, and muscle function improvement; systemic injections restored dystrophin production in the heart and skeletal muscles	2016 Nelson et al. [46]
SpCas9, SaCas9	Single exon deletion	*Dmd* introns 22 and 23 (ex23 del.)	*mdx* mice, *mdx* satellite cells	AAV9 delivery (i.m., i.p., i.v.)	Dual-vector (Cas9 and gRNAs on separate constructs) had higher cutting efficiency than a single-vector system (Cas9 and gRNAs on the same construct) in vitro; dystrophin restoration >10% observed in the heart and skeletal muscles upon systemic treatment; correction also possible in satellite cells	2016 Tabebordbar et al. [47]
SpCas9	Hybrid exon formation via internal exon deletion	*DMD* exons 50 and 54	immortalized DMD patient muscle cells (ex51–53 del.), hDMD/*mdx* mice	Lipotransfection (in vitro)/ electroporation (in vivo)	Dystrophin restoration successful in vitro by WB, not shown in vivo; hybrid exon formation thought to preserve dystrophin rod domain structure better	2016 Iyombe-Engembe et al. [48]
SpCas9	NHEJ reframing, single/multiple exon deletion	*DMD* exons 51, 53, introns 52 and 53 (ex53 del.), 43 and 54 (ex44–54 del.)	immortalized DMD patient muscle cells (ex48–50, or 45–52 del.)	Sequential LV then AdV transduction/AdV transduction	Study showed the possibility of combining both TALEN and CRISPR approaches in one gene editing strategy; also, comparable editing was obtained with Cas9 and gRNA delivered either together or separately in AdV	2016 Maggio et al. [49]
SpCas9	Multiple exon deletion	*Dmd* introns 20 and 23 (ex21–23 del.)	*mdx* mice	Electroporation/AdV transduction	Treatment restored proper calcium dynamics in muscle (electroporation), and restored dystrophin to 50% of wild-type levels, as well as dystrophin-associated complex sarcolemmal localization and muscle membrane integrity (transduction)	2016 Xu et al. [50]
SpCas9	Multiple exon deletion	*DMD* introns 44 and 55 (ex45–55 del.)	DMD hiPSCs, hiPSC-derived skeletal and cardiac muscle cells (ex46–51 or 46–47 del., ex50 dup.), immunodeficient NSG-*mdx* mice	Nucleofection	CRISPR-mediated deletion of the large exon 45–55 region achieved, restored membrane function and dystrophin, β-dystroglycan expression by WB and IHC; mice transplanted with hiPSC-derived skeletal muscle cells showed dystrophin-positive fibers by IHC	2016 Young et al. [42]
SpCas9	NHEJ reframing, single/multiple exon deletion	*DMD* exons 51, 53 (for reframing) introns 52 and 53 (ex53 del.), introns 43 and 54 (ex44–54 del.)	immortalized DMD patient muscle cells (ex48–50, or 45–52 del.)	AdV transduction	AdV with 2gRNA-SpCas9 constructs work as good as those with 1gRNA-SpCas9 constructs in terms of corrective ability and dystrophin restoration	2016 Maggio et al. [51]
SpCas9, SaCas9	Multiple exon deletion, HDR exon correction	*Dmd* exon 53, introns 51 and 53 (ex52–53 del.)	*mdx4cv* mice (nonsense ex53 mutation)	AAV6 delivery (i.m., i.v.)	Dual vector approach (SpCas9 and gRNA separate) yielded higher correction efficiency than single vector approach (SaCas9 and gRNA together); systemic treatment restored dystrophin expression in the heart (~34% dystrophin-positive fibers) and skeletal muscles (~10–50% dystrophin-positive fibers)	2017 Bengtsson et al. [52]
LbCpf1, AsCpf1	NHEJ reframing, single exon skipping, HDR exon correction	*DMD* exon 51, intron 50	DMD hiPSCs, hiPSC-derived cardiac muscle cells (ex48–50 del.), *mdx* mice	Nucleofection (in vitro)/ 1-cell embryo injection (in vivo)	Cpf1 editing successfully restored dystrophin expression and improved mitochondrial function in cardiomyocytes; 5/24 pups (injected at the embryo stage) showed HDR correction and had ameliorated dystrophic phenotypes	2017 Zhang et al. [53]
SpCas9	Duplicated exon removal	*DMD* exon 2, intron 2	immortalized DMD patient muscle cells (ex2 dup.)	PEI transfection/LV transduction	Use of a single gRNA can delete a duplicated exon, resulting in slight dystrophin rescue by WB and IHC	2017 Lattanzi et al. [54]
SpCas9	HDR exon correction	*Dmd* exon 23	*mdx* mice, *mdx* satellite cells	Lipotransfection (template, gRNA), AdV transduction (Cas9)/AdV transduction	Higher transduction efficiency obtained when AdVs were used for both Cas9 and gRNA-HDR template delivery; mice transplanted with corrected satellite cells showed dystrophin-positive fibers by IHC	2017 Zhu et al. [55]
SpCas9	Multiple exon deletion	*DMD* introns 44 and 55 (ex45–55 del.)	humanized *mdx* mice with *DMD* exon 45 del.	Electroporation	Exon 45–55 deletion by CRISPR possible in vivo; first use of the humanized DMD mouse model with exon 45 del. for CRISPR studies	2017 Young et al. [56]
SpCas9	Multiple exon deletion	*DMD* introns 2 and 7 (ex3–9 del.), introns 5 and 7 (ex6–7 del.), introns 6 and 11 (ex7–11 del.)	DMD hiPSCs, hiPSC-derived cardiac muscle cells (ex8–9 or ex3–7 del.)	Nucleofection	Dystrophin with ex7–11 del. showed the least functionality, while those with ex3–9 del. had the highest functionality in terms of assessing iPSC-derived cardiomyocyte calcium cycling	2017 Kyrychenko et al. [57]
SpCas9	HDR correction	*Dmd* exon 23	*mdx* primary muscle cells, *mdx* mice	CRISPR-Gold nanoparticles (i.m.)	5.4% HDR correction of the *Dmd* mutation in *mdx* was observed after CRISPR treatment and cardiotoxin injection, dystrophin-positive fibers found by IHC; 0.8% HDR correction observed without cardiotoxin co-injection, which led to significantly improved hanging test performance	2017 Lee et al. [58]
SpCas9	NHEJ reframing, single exon skipping	*Dmd* exon 51	mice with *Dmd* exon 50 del.	AAV9 delivery (i.m., i.p.)	Successful dystrophin restoration in the heart and skeletal muscles; systemic injections led to improved muscle function; first application of CRISPR in the ex50 del. mouse model	2017 Amoasii et al. [59]
SpCas9	Single exon deletion	*Dmd* introns 50 and 51 (ex51 del.)	primary human skeletal muscle cells	HCAdV delivery	Up to 93.3% exon 51 deletion observed in vitro upon delivery of CRISPR agents by HCAdV	2017 Ehrke-Schulz et al. [60]
SpCas9	NHEJ reframing, exon skipping	*DMD* exon 51, introns 47, 50, 54	DMD hiPSCs, hiPSC-derived cardiac muscle cells (ex48–50 del., pseudo-ex47, ex55–59 dup.)	Nucleofection	All strategies corrected the respective patient mutations and restored dystrophin production in iPSC-derived cardiomyocytes; 3D-engineered heart muscle produced from treated iPSC-derived cardiomyocytes showed improved contractile force	2018 Long et al. [61]
CjCas9	NHEJ reframing	*Dmd* exon 23	mice with deletions in *Dmd* exon 23	AAV9 delivery (i.m.)	CjCas9 displayed higher targeting specificity than SpCas9; use of CjCas9-based CRISPR can lead to successful dystrophin restoration and improvement in muscle function as well	2018 Koo et al. [62]
SaCas9	Hybrid exon formation via multiple exon deletion	*DMD* exons 47 and 58	DMD skeletal muscle cells (ex51–53 del., ex49–50 del., ex51–56 del., ex50–52 del.), humanized *mdx* mice with *DMD* ex52 del.	LV transduction (in vitro)/AAV9 delivery (in vivo; i.v.)	gRNAs designed to produce exon deletions that best preserved dystrophin protein structure were able to show dystrophin restoration in vitro and in vivo (slight rescue in the heart)	2018 Duchêne et al. [63]
SpCas9	NHEJ reframing, exon skipping	*Dystrophin* exon 51	deltaE50-MD canine model (ex50 del.)	AAV9 delivery (i.m., i.v.)	First published study on dystrophin gene correction in a dog model; ~3–70% dystrophin restoration of healthy levels in skeletal muscles and ~92% in the heart found by WB	2018 Amoasii et al. [64]
nSpCas9-ABE7.10	Base editing to correct a nonsense mutation	*Dmd* exon 20	mice with a nonsense mutation in *Dmd* exon 20	trans-splicing AAV2/9 delivery (i.m.)	~3.3% base editing frequency achieved 8 weeks post-treatment with no detectable off-target effects; ~17% dystrophin-positive fibers and restored localization of nNOS observed by IHC	2018 Ryu et al. [65]
dSa/SpCas9-TAM	Base editing to induce exon skipping	*DMD* intron 50 5′ splice site	DMD hiPSCs, hiPSC-derived cardiac muscle cells (ex51 del.)	Lipotransfection	~100% base editing efficiency achieved; corrected iPSC-derived cardiomyocytes had restored dystrophin protein, low CK and miR-31 levels, and restoration of β-dystroglycan expression	2018 Yuan et al. [66]

Abbreviations: NHEJ, non-homologous end joining; HDR, homology-directed repair; ex, exon; NSG, NOD *scid* IL2R gamma; hiPSC, human induced pluripotent stem cells; LV, lentivirus; AAV, adeno-associated virus; AdV, adenovirus; HCAdV, high-capacity adenoviral vector; PEI, polyethyleneimine; i.m., intramuscular; i.p., intraperitoneal; i.v., intravenous; WB, Western blot; IHC, immunohistochemistry; TALEN, transcription activator-like effector nuclease; nNOS, neuronal nitric oxide synthase; CK, creatine kinase; gRNA, guide RNA.
